# Host-Specific Interactions with Environmental Factors Shape the Distribution of *Symbiodinium* across the Great Barrier Reef

**DOI:** 10.1371/journal.pone.0068533

**Published:** 2013-07-03

**Authors:** Linda Tonk, Eugenia M. Sampayo, Scarla Weeks, Marites Magno-Canto, Ove Hoegh-Guldberg

**Affiliations:** 1 ARC Centre of Excellence for Coral Reef Studies and School of Biological Sciences, The University of Queensland, St. Lucia, Queensland, Australia; 2 Biophysical Oceanography Group, School of Geography, Planning and Environmental Management, The University of Queensland, St. Lucia, Queensland, Australia; 3 Global Change Institute, The University of Queensland, St. Lucia, Queensland, Australia; King Abdullah University of Science and Technology, Saudi Arabia

## Abstract

**Background:**

The endosymbiotic dinoflagellates (genus *Symbiodinium*) within coral reef invertebrates are critical to the survival of the holobiont. The genetic variability of *Symbiodinium* may contribute to the tolerance of the symbiotic association to elevated sea surface temperatures (SST). To assess the importance of factors such as the local environment, host identity and biogeography in driving *Symbiodinium* distributions on reef-wide scales, data from studies on reef invertebrate-*Symbiodinium* associations from the Great Barrier Reef (GBR) were compiled.

**Methodology/Principal Findings:**

The resulting database consisted of 3717 entries from 26 studies. It was used to explore ecological patterns such as host-specificity and environmental drivers structuring community complexity using a multi-scalar approach. The data was analyzed in several ways: (i) frequently sampled host species were analyzed independently to investigate the influence of the environment on symbiont distributions, thereby excluding the influence of host specificity, (ii) host species distributions across sites were added as an environmental variable to determine the contribution of host identity on symbiont distribution, and (iii) data were pooled based on clade (broad genetic groups dividing the genus *Symbiodinium*) to investigate factors driving *Symbiodinium* distributions using lower taxonomic resolution. The results indicated that host species identity plays a dominant role in determining the distribution of *Symbiodinium* and environmental variables shape distributions on a host species-specific level. SST derived variables (especially SSTstdev) most often contributed to the selection of the best model. Clade level comparisons decreased the power of the predictive model indicating that it fails to incorporate the main drivers behind *Symbiodinium* distributions.

**Conclusions/Significance:**

Including the influence of different host species on *Symbiodinium* distributional patterns improves our understanding of the drivers behind the complexity of *Symbiodinium*-invertebrate symbioses. This will increase our ability to generate realistic models estimating the risk reefs are exposed to and their resilience in response to a changing climate.

## Introduction

The global decline of coral reefs has generated a broad interest in the widespread symbiosis of reef invertebrates with dinoflagellates of the genus *Symbiodinium.* Hard corals and other invertebrates (e.g. soft corals, giant clams, anemones etc.) that live in symbiosis with *Symbiodinium* typically maintain an obligate relationship, whereby symbionts reside inside their hosts’ endodermal layer. Under prolonged stress conditions the loss of symbionts from the host (coral bleaching) can cause mortality of entire coral colonies across very large reef areas almost simultaneously [1]. Despite the clear importance of *Symbiodinium* in the survival of the holobiont (host plus symbionts), *Symbiodinium* genetic identity has rarely been used to model the risk of reef deterioration or their ability to maintain key functional processes under stress by resisting or adapting to change [2], [3]. The complexity of the *Symbiodinium*-invertebrate symbiosis and the drivers involved in *Symbiodinium* distributions on reef-wide scales lie at the base of this deficit. Various studies have shown that *Symbiodinium* play a key role in the ecology and physiology of specific host species [4], [5], [6], whereby symbiont niche specialization allows the host to inhabit a broader environmental range [7]. On a community level, symbiont-host specificity as well as biogeography underlie distributional patterns [8], [9]. These patterns are mostly studied at the scale of several reefs. Here we study these patterns using host-symbiont data from 68 locations across the Great Barrier Reef (GBR).

Taxonomic knowledge of both the host and symbionts is required to understand how established holobiont species distribution ranges may change in response to future environmental changes [10], [11], [12], [13]. One of the difficulties in using data from different studies on *Symbiodinium* is that different markers have been used to identify genetically distinct *Symbiodinium* (e.g. 18s rRNA, internal transcribed spacer region (ITS) 1 and 2, large ribosomal subunit region (LSU) D1/D2, chloroplast 23S rDNA). Not only does this limit direct comparison, but it also forms an obstacle since different genetic markers provide different levels of taxonomic resolution.

At present, the genus *Symbiodinium* is divided into nine broad genetic clades: clades A-I [14], [15], of which most contain various genetically and ecologically distinct types [5], [7], [16], [17], [18]. Several *Symbiodinium* types have been described using well-established phylogenies with functional and ecological differences. While some of these are described as species, the majority remains undefined [19], [20], [21]. Physiological differences between *Symbiodinium* types include differences in photosynthetic performance [22], cell size [23], pigment composition [24], and tolerance to heat stress [6], [10], [22], [25], [26], [27], [28]. While identification at the clade level may, at times, suffice to explain spatial patterns in symbiont communities, the exclusion of intra-cladal differences more often obscures ecological patterns in *Symbiodinium* distribution. Examples of this can be found across several coral genera that have distinct intra-cladal zonation of *Symbiodinium* types over depth, i.e. *Madracis* [4], [29], [30], *Stylophora*, *Pocillopora* [7] and *Seriatopora* [31]. In addition, techniques used to identify *Symbiodinium* can differ in sensitivity. Generic PCR techniques pick up the dominant (more than 5–10%) *Symbiodinium* whereas quantitative PCR (qPCR) also detects background types that occur at much lower abundance [32], [33].

Using currently available ecological and physiological information on reef-invertebrate associations with *Symbiodinium* to make meaningful predictions on the relative vulnerability of coral reefs, requires extending analyses to community and reef wide scales. Consequently, there is a desire to include various non-scleractinian reef-dwelling invertebrates that also live in symbiosis with *Symbiodinium* such as Alcyonacea (soft corals), Actinaria (anemones), Milleporina (fire corals), Hadromerida (sponges) and Veneroida (giant clams). Factors that are known to influence *Symbiodinium* distribution include: host identity and specificity (e.g. certain symbiont types are only found in specific host species or genera) [4], [5], [9], [18], [34], [35], longstanding biogeographic partitioning [5], [9], [11], regional and local environmental conditions (e.g. light, [4], [22] temperature, [4], [9], [10], [36], and turbidity [9], [35], [36]). On a community level, the intertwined processes mentioned above shape the diversity of symbioses found across reefs.

The Great Barrier Reef (GBR) is the world’s largest continuous reef system, spanning approximately 2300 km, including 10% (approximately 3000 reefs) of coral reefs worldwide and comprising a large number of environmentally distinct areas that lack the presence of large biogeographic barriers [37]. It is also one of the most densely studied areas worldwide in terms of reef invertebrate symbioses with *Symbiodinium* (at least 28 studies, see references in methods). Given these attributes, we use the GBR as a model system to investigate how long standing environmental and evolutionary factors drive symbiotic communities.

## Materials and Methods

### Database Compilation

Data were compiled from published literature (up to 2012) on endosymbiotic *Symbiodinium* from the Great Barrier Reef (GBR), including only non-experimentally treated data and from pre-bleaching data collections [5], [7], [11], [12], [18], [25], [26], [31], [34], [38], [39], [40], [41], [42], [43], [44], [45], [46], [47], [48], [49], [50], [51], [52], [53], [54] (also see [55] for SymbioGBR, the web-based version of this database). The database only includes information obtained with techniques that focus on the dominant *Symbiodinium* types in the resident symbiont population of the host, excluding information on background *Symbiodinium* obtained with qPCR (e.g. [36], [56]).

**Figure 1:**
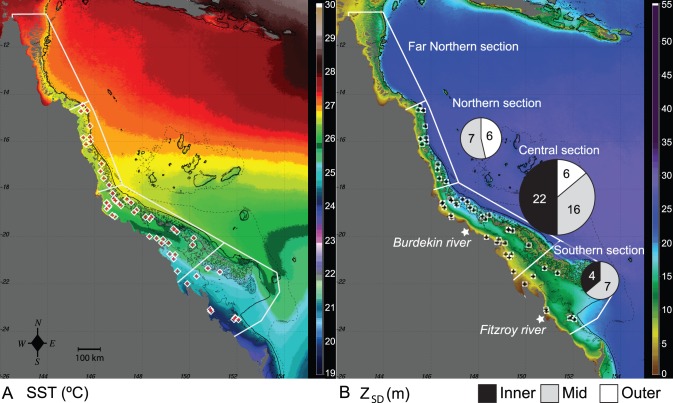
MODIS satellite images for the Great Barrier Reef. Long-term sea surface temperature climatology (SST, °C) (A) and Secchi depth climatology (Z_SD_, m) (B). Sites are indicated with crosses. Pie charts represent shelf position (inner, mid and outer) of collection sites for each section of the Great Barrier Reef.

Each individual host was entered as a data point and annotated with collection date, site, shelf position, GPS position, collection depth (m), distance to the coastline (km), various sea surface temperature (SST) and turbidity indicators (Z_SD_, for details see environmental section), host identity (order, family, genus, and species if available), and symbiont identity (clade and ‘ITS-type’). From a total of 3833 entries, those entries containing missing data in analytical fields were removed. The final effective database consisted of 3717 entries from 68 locations (see Table S1) situated in three of the four sections that divide the GBR: the far northern section (no sites included), the Cairns/Cooktown or northern section, the Townsville/Whitsunday or central section, and the Mackay/Capricorn or southern section (Figures 1A and B).

### *Symbiodinium* Nomenclature

The database contained taxonomic information on *Symbiodinium* identity from various molecular markers (18S rDNA, D1/D2 LSU rDNA, cp23S rDNA, ITS1 and ITS2) analyzed with different techniques (restriction fragment length polymorphism [RFLP], single-strand conformation polymorphism [SSCP] and denaturing gradient gel electrophoresis [DGGE]). The database therefore differed in the taxonomic level of resolution for *Symbiodinium* [20] and in the use of dissimilar nomenclatures (namely ITS1 and ITS2, see Table S2). For comparative purposes and because the widest diversity of host species entries in the database were in the ITS2-type format (ITS2 from 188 host species covering 1607 entries, ITS1 from 22 species covering 1984 entries), ITS1 entries (*sensu* van Oppen) were cross-referenced to the ‘ITS2-type’ nomenclature (*sensu* LaJeunesse) using information that was available for both the ITS1 and ITS2 region [20], [49], [57] (see Table S2). Note that for some entries this may lead to loss of resolution. The original 3717 entries in the database were used for analysis using the broader clade level designation while analysis on the symbiont ITS-type level was possible for 3597 of these entries from 63 sites.

### Host Community

Hard corals, octocorals and other *Symbiodinium* harboring reef invertebrates were included in the analysis to obtain an apt representation of the wide diversity of the symbiotic community. Recovered species richness of local *Symbiodinium* communities is expected to be an asymptotic curve leading to a plateau proportional to the sampling intensity of the invertebrate host community (although this is also dependent on the technique used to identify subgroups within *Symbiodinium*; the plateau will be reached sooner when using a lower taxonomic resolution). Assembled database information was not standardized for sampling design across locations. In order to calculate the extent to which the ‘sampled host species diversity’ within the database assembled here provided an accurate representation of ‘locally present host community diversity’, publicly available host species diversity data were sourced from the Australian Institute of Marine Science (AIMS), 2012, for octocorals (http://e-atlas.org.au/content/octocorals-great-barrier-reef-0) and reef-building coral communities (http://e-atlas.org.au/content/hard-coral-biodiversity-surveys-gbr, both accessed on 19/06/2012, see Table S1). This was done for 60 out of 68 sites in the database, for which publicly available host species diversity data were available. The following equation describes the percentage of species present in the database assembly compared to the locally present number of scleractinian and octocoral species: % representation = # species in the database/# locally present scleractinian+zooxanthellate octocoral species. Separately, an accumulation curve was plotted of host genera versus symbiont types per database site and reference data were added from *Symbiodinium* diversity studies in the Pacific Ocean (various sites from the central GBR, southern GBR, Japan, Hawaii, Zanzibar, and Thailand [5], [9], [11], [16]; Lizard Island [Tonk et al. unpublished data]).

### Environmental Parameters

Sea surface temperature (SST) and turbidity measures (Secchi depth, Z_SD_) were derived from the Moderate Resolution Imaging Spectroradiometer (MODIS) aboard the National Aeronautics and Space Administration (NASA) Terra and Aqua satellites (modis.gsfc.nasa.gov). Time series were generated at 1 km spatial resolution for the periods 2000–2009 and 2002–2009 for SST and Z_SD_, respectively (Figure 1A and B). The SST metrics included the monthly mean climatology over the nine-year period as well as the long-term (nine-year) SST climatology for each of the 68 sites in the database. Similarly, the standard deviation of the long-term climatology over the period of 2000–2009 (SSTstdev) was calculated from the monthly climatology over the nine-year period for each site and used as a proxy for the range of SST data the holobiont is exposed to. The Z_SD_ metrics were determined using a GBR-validated algorithm generated by matching the 10% photic depth level (Zeu_10%_) against GBR Secchi data (1997–2010) [58], [59]. Z_SD_ metrics included the monthly mean Z_SD_ climatology over the entire period as well as the long-term (seven-year) overall mean Z_SD_ for the same sites as the SST data. For optically shallow locations (e.g., shallow outer reefs) where Zeu_10%_ may exceed the actual physical depth resulting in bottom contamination of the photic depth signal, an alternative pixel was manually selected [59].

### Statistical Analysis

**Figure 2:**
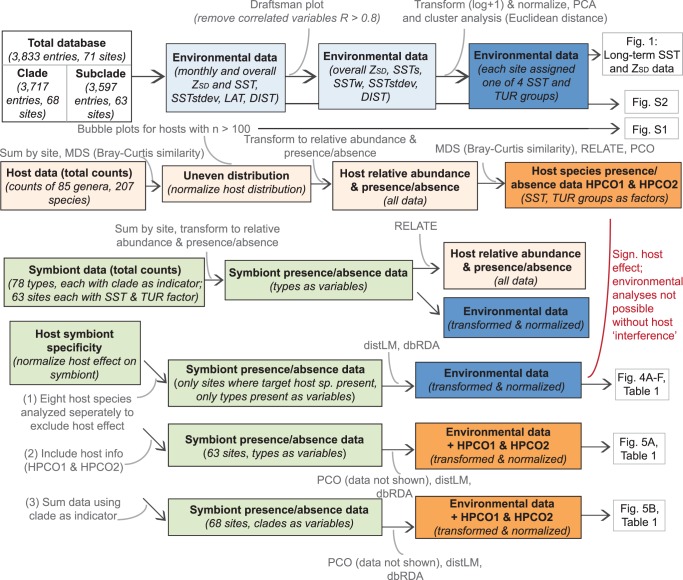
Flowchart of statistical analyses performed in PRIMER with PERMANOVA add-on.

Multivariate analyses and regression analyses were performed in PRIMER-e (v6.1.13) with the PERMANOVA add-on (v1.03; [60]). The database consisted of two species diversity matrices, one for the host and one for the associated symbionts, as well as a matrix of environmental parameters linked to each of the sites. Details for analyses of each are specified separately below and integration of the various levels is visualized in a flowchart (Figure 2).

The environmental dataset was analyzed using draftsman plots and Pearson correlation coefficients to calculate inter-correlation amongst variables and remove redundancy in the dataset (Pearson’s *r* >0.8 or *r*<-0.8), prior to subsequent analyses. Draftsman plots revealed a significant inter-correlation between monthly mean SST data from the summer and winter months. To limit redundancy, the two warmest (January and February) and coldest (July and August) months were averaged and used instead of all individual monthly averages. In subsequent analyses these values were used as temperature variables for summer (SSTs) and winter (SSTw) respectively. SSTs and SSTw further showed a high correlation with long-term mean SST as well as latitude, both of which were omitted from subsequent analyses. Inter-correlations were present between the long-term and all monthly Z_SD_ means (Pearson’s *r* >0.8) and only the long-term mean Z_SD_ was maintained for subsequent analyses. The remaining environmental variables SSTs, SSTw, SSTstdev, long-term mean Z_SD_ and distance from the coast were transformed (log+1) and normalized. A Principal Component Analyses (PCA) was done to visualize spatial patterns between sites as a function of environment, and these were used to assign each site into one of four environmentally informative groups for SST and for Z_SD_ independently (SST1 and TUR1 being the lowest group, see Table S1 for categories). Note that these groupings were only used for downstream illustrative purposes. For statistical analyses original environmental data were used for the non-redundant variables as stated above.

The host dataset was analyzed independently to determine whether, due to uneven sampling design across the different studies, host community assemblages differed significantly across sites and/or were related to environmental data. At two of the sites, Hazelwood and Double Cone Island, only a single specimen was collected from the octocorals *Junceella* sp. and *Euplexaura* sp. respectively. Since no other records were present for these species in the database, these entries formed significant outliers and were excluded from further analyses. The sampling of host organisms was highly uneven across sites (see Figure S1 for the spread of eight heavily sampled host species depicted as Multi-Dimensional Scaling [MDS] bubble plots). The host data were therefore transformed to relative species abundance per site (number of entries per species per site divided by the total number of entries per site) to reduce the effect of unstandardized sampling effort. Similarity was calculated using Bray-Curtis and an MDS was plotted to visualize the spatial ordination of sampled host communities (data not shown). RELATE was used to test for a relation between the host and the environment and between the host and the symbiont data matrices. RELATE tests the hypothesis of no relation between multivariate patterns from two datasets by calculating a rank coefficient (Spearman’s rho) between similarity matrices of the sample sets (rho ≈ 0 indicates no relation is found, rho = 1 indicates a perfect relation).

To determine whether environmental factors drive *Symbiodinium* spatial distributions across the GBR, while taking into consideration that host communities are unequally distributed and host-symbiont specificity plays an important role (RELATE, p = 0.001, rho = 0.336 between host and symbiont data), three approaches were used to reduce dimensionality in the dataset. These approaches were to: (a) exclude host effects by analyzing symbiont data for host species with over 100 samples in the entire database (*Acropora millepora*, n = 598; *Stylophora pistillata*, n = 517; *Pocillopora damicornis*, n = 509; *Seriatopora hystrix*, n = 515; *Sinularia flexibilis*, n = 309; *A. tenuis*, n = 227; *Turbinaria reniformis*, n = 228 and *A. valida*, n = 157), (b) add the first two principle coordinate axes of a Principle Coordinate Analyses (PCO) on the host presence/absence data (HPCO1 and HPCO2 for continued reference) to the environmental data matrix, and hereby incorporate 48% of the variation explained in the host data as covariates in subsequent linear regression data analyses and (c) sum the data using clade as an indicator to reduce the effect of host-symbiont specificity on the type level.

The three approaches outlined above were used to perform both multivariate and linear regression analyses on the *Symbiodinium* data after transformation to a presence/absence data matrix to reduce effects of unequal sampling intensities. Distance-based analysis on a linear model (distLM) was used to model the relationship between symbiont dissimilarity data and environmental variables, which included the host as a co-variate (HPCO1, HPCO2). Marginal tests assessed the importance of each variable separately. In the sequential tests a forward search was used to find the optimal fit based on an adjusted R^2^ (proportion of explained variation for the model) by sequentially adding environmental variables. The pseudo-*F* statistic was used to test the general null hypothesis of no relationship, in which the P-value provides the significance level and the percentage of variance explained is shown per environmental variable. The data were visualized with distance based redundancy analyses plots (dbRDA), which are generally used to perform an ordination of fitted values from a given model. In a dbRDA plot the first two axes are shown which represent the highest percentage of explained variation out of the fitted model and the total variation. Percentage of fitted variation specifies the variability in the original data explained by the fitted model and percentage of total variation specifies the variation in the fitted matrix. Vector overlays using the environmental data and symbiont data separately as predictor variables (drawn as multiple partial correlations) were applied to visualize the effect, strength and direction of the different variables in the ordination plots.

## Results

### Environmental Data

The database includes information on reef invertebrate symbioses with *Symbiodinium* from 68 sites across the Great Barrier Reef (GBR) (Figure 1A). The majority of these sites, 44, were located in the central section while sampling effort on inshore- and outer reef locations of the northern (far northern and Cairns/Cooktown) and southern section of the GBR was low (Figure 1B, Table S1). The PCA explained 87% of the variation in the environmental data and a strong spatial distribution of the SST groups was evident with increasing SSTs and SSTw across the latitudinal gradient (Figure S2A). Turbidity groups were distributed according to Z_SD_ and SSTstdev but interestingly showed no direct correlation with distance from shore (Figure S2B). Sites in the inshore-situated Whitsunday Islands and Magnetic Island Group formed distinct clusters due to high turbidity, the Capricorn Bunker Group sites due to low SST and sites in the Keppel Islands due to high turbidity coupled with low SST.

### Host Community

**Figure 3:**
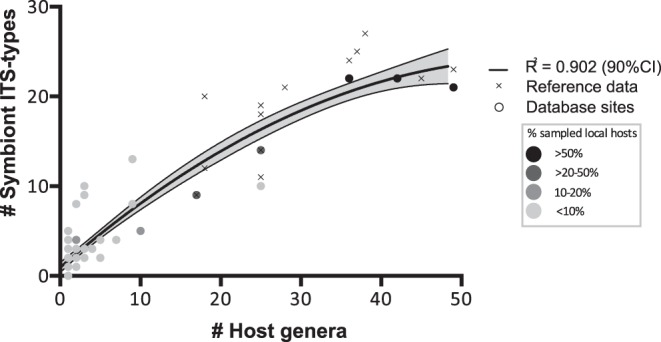
Accumulation curve of *Symbiodinium* ITS-types versus host genera. Database sites (circles) and reference data (crosses) are included. The shaded area indicates the 90% confidence interval and the circles increasing grey intensity indicates percentage of local hosts sampled.

The database entries include 207 host species that were mainly Scleractinia (reef-building corals, 86%) with a range of other hosts from the Alcyonacea (soft corals), Actinaria (anemones), Milleporina (fire corals), Hadromerida (sponges), Zoantharia, Corallimorpharia, Hydroida, Helioporacea (blue corals) and Veneroida (giant clams). The percentage of sampled hosts represented in the database compared to locally present host species diversity highlighted that overall sampling efforts were exceedingly poor at each site. Only 17 of the 60 sites had over 5% of the locally present host community diversity examined for their *Symbiodinium* identity and at only three of these sites the percentage of sampled host species diversity exceeded >50% (Table S1). The accumulation curve of the relationship between host sampling effort and recovered symbiont species diversity showed that most sampling sites fail at describing locally present community diversity. This data clearly reflected a deficiency in our current knowledge of *Symbiodinium* diversity across host genera in an area such as the GBR where sampling intensity is seemingly high (Figure 3).

The non-transformed abundance data of the eight most heavily sampled host species (Figure S1) as well as the lack of a significant relation between the host and environmental data matrix (RELATE, p = 0.244, rho = 0.029) indicated that spatial patterns in host diversity were the result of an unequal sampling design rather than environmental differences between study sites.

### Descriptive Data of *Symbiodinium* Diversity in Coral and Non-coral Hosts

Five *Symbiodinium* clades were found across the GBR (clade A, B, C, D, and G). The vast majority of hosts (92%) contained clade C *Symbiodinium* only, 1.5% clade D only whereas 5% contained both clade C and clade D *Symbiodinium*. Approximately 94% of the sampled hosts contained symbionts from a single clade (i.e., just clade A, B, C, D or G) although background types may not be detected if they constitute less than 5–10% of the total population [32], [33]. A significant relationship between the host and symbiont data matrix (RELATE, p = 0.001, rho = 0.336) confirmed that host identity (i.e., host species reported) played an important role in driving *Symbiodinium* community diversity.

Clade C symbionts were found in both octocorals and hard corals, whereas clade D was mainly found in hard corals. Clades B, G, and A were respectively found in 0.08%, 0.16%, and 0.2% of host colonies with clade B symbionts being restricted to octocorals, clade G to octocorals, sponges and found in a single *Stylophora pistillata* colony [12] and clade A in fire corals (*Millepora* spp.) as well as in several acroporids (*Acropora longicyathus*, *A. millepora* and *A. valida*).

The database includes 62 *Symbiodinium* types, of which 56 were clade C types, and the remaining *Symbiodinium* A7 (fire corals), B1, B36 (*Nephthea* spp.) and three clade D types (D1, D1-4 [a.k.a. D1a or *Symbiodinium ‘trenchi’*] and D3; see Table S2). The majority (75%) of *Symbiodinium* types across all clades were highly host specific and restricted to either a single host species or species belonging to one host genus. Examples of host specific symbiont types were: C3i and C3k in the genus *Acropora*, C17 in the genus *Montipora* or C120 in *Seriatopora hystrix.* A remaining 25% were host generalist types such as C1, C3, C3h, C21 and D1-4, which were identified from a range of host species (see Table S2). Some hosts harbored similar symbiont types throughout the latitudinal range (for instance *Sinularia flexibilis*, *Lobophytum compactum* and *Coscinaraea columna* all harbor *Symbiodinium* C1), but many hosts showed some geographical distribution in symbiont associations (*Heteractis magnifica* with *Symbiodinium* C25, C68 and C67 found in respectively the southern, central and northern GBR; *Sarcophyton* sp. with C3j in the southern GBR, C65 found in the central GBR and C1 in the central and northern GBR; *A. nobilis* with *Symbiodinium* C3k in the southern GBR, C3 in the southern and central GBR, C1, C3i and D1 in the northern GBR; and *Fungia fungites* with *Symbiodinium* C21 found in the southern section, C1 in the central section and C3h in the northern section).

### *Symbiodinium* in Single Host Species (Removing the Host Effect)

**Figure 4:**
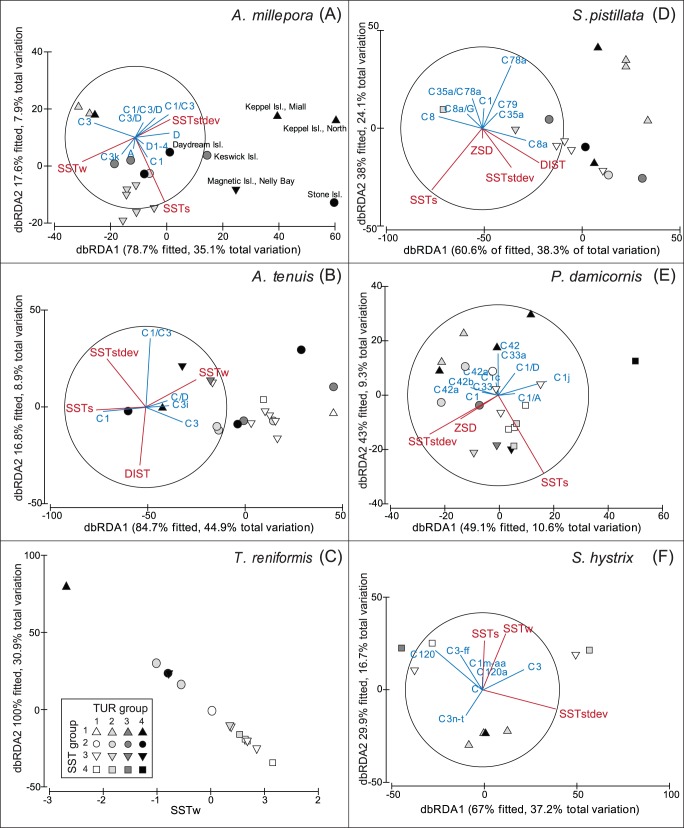
DbRDA ordination relating environmental variables to *Symbiodinium* ITS-types per host species. Presence/absence data of *Symbiodinium* ITS-types in (A) *Acropora millepora*, (B) *Acropora tenuis*, (C) *Turbinaria reniformis,* (D) *Stylophora pistillata*, (E) *Pocillopora damicornis* and (F) *Seriatopora hystrix* at different sites are shown in biplot projections (log transformed and normalized environmental data is shown in red and symbiont ITS-types in blue). For illustrative purposes only, sites are designated a sea surface temperature (SST) group (indicated by symbols) and turbidity (TUR) group (indicated with different shades of grey). The ‘% of fitted’ indicates the variability in the original data explained by the fitted model and ‘% of total variation’ indicates the variation in the fitted matrix.

Eight most commonly sampled host species (Acropora millepora, A. tenuis, A. valida, Stylophora pistillata, Pocillopora damicornis, Seriatopora hystrix, Sinularia flexibilis and Turbinaria reniformis) were investigated separately to understand how the environment influences symbiont distributions without the effect of host specificity driving symbiont differentiation. As S. flexibilis only hosted Symbiodinium type C1 and A. valida was collected at just three locations, these host species are not included in the analysis due to a lack of comparative data. The linear model (distLM on Bray-Curtis dissimilarities of Symbiodinium presence/absence data per host species) explained between 20 to 62% of the total variation in the fitted matrix (i.e. the fitted relation between the symbiont matrix per host species and the explanatory variables) depending on host species investigated (Figure 4A-F, Table 1). While 20% of total variation explained in the fitted model is low, indicating that up to 80% of the variation found in the data matrix remains unexplained, 62% of total variation explained by the environmental parameters tested is a good indication that most relevant environmental parameters are included in the analyses since a large part of the variation in symbiont data is explained by the model.

**Table 1:** Summary of distLM analyses.

Marginal tests				Sequential tests						
Group	Pseudo -F	P	% Variance explained	Group	Cumulative adjusted R^2^	Pseudo-F	P	% Variance explained	% Cumulative variance	df
***A. millepora***										
DIST	6.2	0.002*	26.8	SSTstdev	0.2	6.6	0.002*	28.0	28.0	17
ZSD	2.2	0.073	11.4	SSTs	0.3	2.0	0.095	8.0	36.0	16
SSTs	1.5	0.185	8.3	SSTw	0.3	2.3	0.083	8.6	44.6	15
SSTw	4.6	0.005*	21.4							
SSTstdev	6.6	0.002*	28.0							
***A. tenuis***										
DIST	2.5	0.139	12.7	SSTstdev	0.2	6.4	0.017*	27.3	27.3	17
ZSD	1.5	0.236	8.1	DIST	0.2	1.4	0.232	6.0	33.2	16
SSTs	1.6	0.221	8.7	SSTs	0.3	2.2	0.164	8.6	41.9	15
SSTw	2.3	0.156	11.8	SSTw	0.4	3.4	0.102	11.2	53.1	14
SSTstdev	6.4	0.024*	27.3							
***T. reniformis***										
SSTw	5.8	0.002*	30.9	SSTw	0.3	5.8	0.005*	30.9	30.9	13
DIST	0.2	0.86	1.6							
ZSD	3.9	0.024*	23.3							
SSTs	3.7	0.037*	22.2							
SSTstdev	4.0	0.031*	23.6							
***S. pistillata***										
DIST	2.7	0.056	17.4	SSTs	0.3	5.8	0.002*	31.0	31.0	13
ZSD	0.2	0.923	1.4	DIST	0.3	2.6	0.114	12.5	43.4	12
SSTs	5.8	0.003*	31.0	SSTstdev	0.5	4.4	0.044*	16.2	59.6	11
SSTw	2.1	0.14	13.7	ZSD	0.5	1.0	0.356	3.7	63.3	10
SSTstdev	0.3	0.706	2.6							
***P. damicornis***										
DIST	0.4	0.762	2.1	SSTs	0.0	2.0	0.113	9.4	9.4	19
ZSD	0.5	0.738	2.4	SSTstdev	0.1	1.3	0.253	6.2	15.6	18
SSTs	2.0	0.124	9.4	ZSD	0.1	1.3	0.257	6.0	21.6	17
SSTw	1.2	0.307	6.0							
SSTstdev	1.2	0.294	6.2							
***S. hystrix***										
DIST	1.6	0.209	19.1	SSTstdev	0.1	2.2	0.144	24.0	24.0	7
ZSD	0.9	0.492	11.6	SSTw	0.3	2.3	0.14	21.3	45.3	6
SSTs	1.3	0.251	16.0	SSTs	0.3	1.2	0.306	10.3	55.6	5
SSTw	1.5	0.219	18.1							
SSTstdev	2.2	0.137	24.0							
**ITS2-type**										
DIST	2.2	0.049*	3.4	HPCO1	0.2	14.0	0.001*	18.7	18.7	61
ZSD	1.2	0.304	1.9	HPCO2	0.2	4.5	0.002*	5.7	24.3	60
SSTs	3.1	0.019*	4.9	DIST	0.2	2.5	0.039*	3.0	27.4	59
SSTw	1.9	0.094	3.0	SSTstdev	0.2	1.7	0.135	2.0	29.4	58
SSTstdev	2.0	0.08	3.1	SSTw	0.3	1.8	0.103	2.2	31.6	57
HPCO1	14.0	0.001*	18.7	SSTs	0.3	3.6	0.008*	4.2	35.8	56
HPCO2	3.7	0.013*	5.7							
**Clade**										
HPCO1	4.0	0.018*	5.9	HPCO2	0.1	4.9	0.011*	7.1	7.1	64
HPCO2	4.9	0.006*	7.1	DIST	0.1	5.3	0.005*	7.1	14.2	63
DIST	4.2	0.014*	6.2	HPCO1	0.2	4.5	0.02*	5.8	20.1	62
SSTstdev	2.5	0.086	3.8							
SSTs	−0.2	0.982	−0.4							
SSTw	1.1	0.338	1.8							
ZSD	1.1	0.343	1.7							

Table shows output for model selection of the relationship between *Symbiodinium* communities, host and/or environmental variables: per host species (*Acropora millepora*, *A. tenuis*, *Turbinaria reniformis, Stylophora pistillata*, *Pocillopora damicornis* and *Seriatopora hystrix*), of all host species combined at ITS2-type level and at clade level.

* Significant values.

#### Acroporidae

The environmental parameters significantly influenced symbiont distributions in *A. millepora* (RELATE, p = 0.001, rho = 0.513) and *A. tenuis* (RELATE, p = 0.02, rho = 0.215). A total of 43 and 54% of the variation in the fitted model was explained by the distribution of *Symbiodinium* types for *A. millepora* and *A. tenuis* respectively (Figures 4A and B). The sequential test (assessing the contributing effects of the variables combined by fitting them as covariates) was significant for SSTstdev for both acroporids respectively explaining 28 and 27.3% of the variation (Table 1). These percentages are relatively high considering the various environmental factors at play. The addition of SSTs and SSTw (and additionally DIST for *A. tenuis*) contributed to the selection of the best model (Table 1). More specifically, in *A. millepora* SSTstdev explained the presence of clade D symbionts in the Keppel Islands (Figure 4A).

#### Turbinaria reniformis

SSTw explained 31% of total variation in the *Symbiodinium* distribution data of *T. reniformis* (Figure 4C). In addition SSTs, SSTstdev and DIST individually influenced symbiont distribution patterns as shown by the marginal tests. However the sequential tests demonstrated that SSTw alone best fitted the linear model (Table 1). *Symbiodinium* C1 was found associated with corals at lower SSTw whereas a change to higher SSTw was accompanied by a clear shift to a combination of C1/D *Symbiodinium* (Figure 4C).

#### Pocilloporidae

In total 62% of the variation in the *Symbiodinium* distribution data of *S. pistillata* was explained (Figure 4D), with 31% of this variation explained by SSTs. Both SSTs and SSTstdev were significant in the sequential test, and the addition of DIST and Z_SD_ contributed to the selection of the best model (Table 1). The environmental variables explored only explained a small percentage of the total variation (20%) in the *Symbiodinium* distribution data of *P. damicornis* (Figure 4E), whereas 54% was explained for the distribution data of *S. hystrix* (Figure 4F). None of the variables significantly influenced each of these species individually or sequentially. In both host species the addition of SSTstdev and SSTs contributed to the selection of the best model but were not found significant, including Z_SD_ and SSTw for *P. damicornis* and *S. hystrix* respectively (Table 1).

None of the environmental factors described *Symbiodinium* distributions across all six species and it was evident from the marginal tests (assessing the importance of each variable separately) that different environmental parameters individually influenced patterns of variation in the *Symbiodinium* community depending on the host species investigated. While a similar trend was seen in the sequential tests, SST derived variables most often fitted the model best or contributed to the selection of the best model (Table 1).

### *Symbiodinium* Across Hosts on the GBR (Host Included as an Environmental Factor)

**Figure 5:**
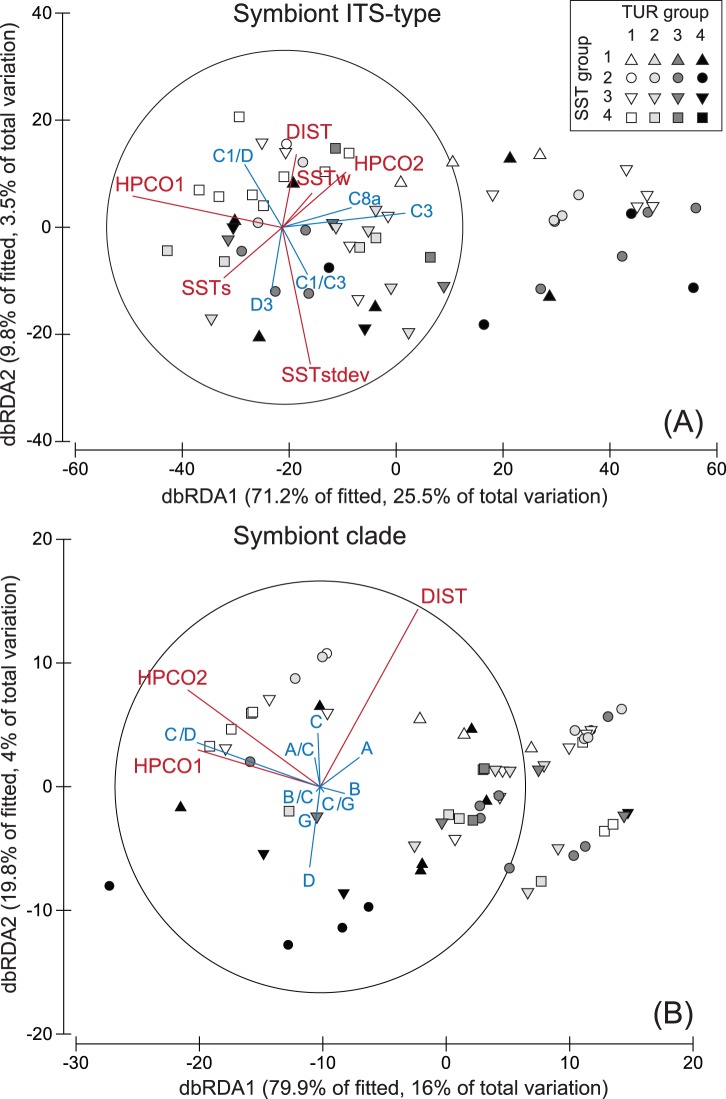
DbRDA ordination relating environmental variables to *Symbiodinium* ITS-type data of all host species combined. Presence/absence data of *Symbiodinium* ITS-types (A) and presence/absence data of *Symbiodinium* summed by clade (B) showing biplot projections (r >0.3) for environmental data (red; including the host variation expressed as HPCO1 and HPCO2) and symbiont types (blue). Sites are designated a sea surface temperature (SST) group (indicated by symbols) and turbidity (TUR) group (indicated with different shades of grey). The ‘% of fitted’ indicates the variability in the original data explained by the fitted model and ‘% of total variation’ indicates the variation in the fitted matrix.

A relationship was found between symbiont type and host species (RELATE, p = 0.001, rho = 0.336) but not between symbiont type and environmental data (dataset including all host species and symbiont types). DistLM on Bray-Curtis dissimilarities of the *Symbiodinium* type presence/absence data (Figure 5A), including the host as an environmental factor only explained 29% of the total variation in the fitted model. The marginal and sequential tests were significant for the same set of variables namely: HPCO1, HPCO2, DIST and SSTs (Table 1). Note that little meaning can be drawn from p-values for individual terms after the first large p-value is encountered in a series of sequential tests [60]. Indeed, no relation with SSTs was discernable from the ordination plot. Sites were somewhat structured in relation to turbidity group with most low turbidity sites situated in the top half of the ordination plot. Distance to shore (DIST) was also related to low turbidity groups. *Symbiodinium* types C1, C3, C8a, D3 and mixtures of types C1/D and C1/C3 showed an, albeit weak, effect as predictor variables in the biplot projections (Figure 5A, r >0.3).

No relationship was found between symbiont clade and environmental or host data. DistLM on Bray-Curtis dissimilarities of *Symbiodinium* clade presence/absence data (Figure 5B, Table 1) showed that the variability was poorly explained (20% total variation). HPCO1, HPCO2 and DIST significantly contributed as individual variables. The sequential tests indicated that the same set of variables best fitted the model, albeit poorly. Although less of the variation in *Symbiodinium* distribution was explained when *Symbiodinium* clade level data were used instead of types, the ordination plot showed a higher level of structuring according to distance from shore (DIST, linked to low turbidity groups).

## Discussion

The distribution of *Symbiodinium* at a community level across the entire GBR has not previously been pursued despite compelling arguments to do so [9], [11], [16], [61]. Here, information was compiled from 26 studies on reef invertebrate-*Symbiodinium* associations on the GBR. Analyses showed that most of these studies focused on only a few host species within defined locations. As a result the host community in which *Symbiodinium* was examined was highly unbalanced across sites and poorly represented the complete community diversity present at each of those sites due to low sampling efforts. An attempt was made to standardize the data and reduce host effects by (i) analyzing frequently sampled host species separately, (ii) including host species distribution as an environmental variable and (iii) using data at a lower taxonomic resolution (by clade) to reduce the effect of host-symbiont specificity. The main outcomes were that host species identity played a dominant role in determining the distribution of *Symbiodinium* and various environmental variables explained additional variability within each of those hosts but the significance of distinct environmental drivers was host specific (i.e. not all hosts are influenced by the same parameter).

### Host Identity and Specificity

The host identity was a primary factor in the distribution of *Symbiodinium*. This was evident from the inclusion of the principal coordinate axes of host distribution data, which increased the total variation explained in the fitted model by 20%, with HPCO1 and HPCO2 explaining 24% of this variability. The host species distribution range can influence the symbiont distribution especially in the case of host-specificity (that is, certain symbionts are only found in a specific host species or genus). For example, in cases where host species are symbiont specific, the absence of the host species means that the symbiont is also not present. However, when a certain host species has formed a relationship with a symbiont that can inhabit a broad number of host species, then the absence of a host does not determine whether or not the symbiont might be found in that location. The level of host-specificity is partially linked to symbiont acquisition strategy, i.e. coral larvae take up symbionts from the environment or from the parent colony [5], [34]. Many *Symbiodinium* types were found to be host or genus specific (e.g. in the genus *Acropora*, *Montipora* and within the family of the pocilloporids) and few *Symbiodinium* types occurred across a wide range of different host species (C1, C3, C3h and C21) [5], [9], [11]. Patterns of host species specificity potentially mask or counteract the influence of environmental variables when multi-variate analyses are applied to the complete *Symbiodinium* dataset covering a wide range of host species. The host can further influence the direct environment or ‘micro-environment’ of the symbiont depending on differences such as colony shape, host tissue thickness and host pigments [62], [63]. Finally, it is also possible that host distributions are driven by their *Symbiodinium* associations. For example, if *Symbiodinium* are important to the overall host tolerance to thermal stress, then the presence or absence of thermally tolerant *Symbiodinium* may determine whether or not the coral host species survives such a stress event [26].

While the importance of host identity in *Symbiodinium* distribution is not surprising or new in itself [4], [5], [9], [18], [35], [36], the clear statistically significant calculation of the relative contribution of host identity found here within a large community based dataset is. Moreover, the discovery of the adaptive response of particular host-symbiont combinations is easily, though incorrectly, extrapolated to the entire coral community. In contrast, the relative ratio of different life history traits across host community assemblages influence spatial and environmental patterns in *Symbiodinium* distributions, highlighting the necessity to integrate life-history traits, plasticity and evolutionary processes.

### Environmental Parameters

On a biogeographical scale, few host species were adequately sampled to perform a separate analysis on the relationship between the symbiont dissimilarity data and the environmental variables. When these host species were analyzed separately, none of the environmental factors unequivocally described *Symbiodinium* distributions. Although specific environmental parameters influenced *Symbiodinium* GBR-wide patterns differently depending on the host species involved, SST derived variables best fitted the model or contributed to the selection of the best model on most occasions (Table 1). Of these variables SSTstdev, either in isolation or in conjunction with other environmental factors, most often significantly contributed to the selection of the best model.

In a recent study on regional symbiont distribution patterns in *Acropora millepora* on the GBR, SST anomaly, mean summer SST, mud, and carbonate content emerged as driving factors which explained 51.3% of the total variation in the symbiont community [36]. These *A. millepora* data were included in our study along with additional *Symbiodinium* type information of *A. millepora* from other locations [5], [11], [12], [18], [25], [34], [52]. Similarly, our results showed SSTstdev as the most important driving factor with SSTs and SSTw contributing to the selection of the best model describing *Symbiodinium* distribution. Thermal environments vary with latitude (at low latitude seasonal differences in temperature are smaller), distance to the coast (changes in inshore SST) and the current flow [64]. Thermal history further influences the bleaching susceptibility of the coral host [65] and adaptation and/or acclimatization potentials may be higher in holobionts that are exposed to more variable environments [66]. The evidence for these assumptions is, however, fragmentary and to a large extent conflicting. Interestingly, SSTstdev was also the most important driving factor in the symbiont distribution of *A. tenuis* while DIST, SSTs and SSTw contributed to the selection of the best model. The re-occurance of SSTstdev as a driving factor strengthens the argument for the importance of SST deviation or anomaly. In addition, these outcomes suggest an overall similarity in the factors driving the distribution of symbionts within species of the same genus.

All SST derived variables and Z_SD_ separately explained *Symbiodinium* distributions in *T. reniformis.* SSTw by itself sufficed in the sequential tests and was linked to a clear shift from *Symbiodinium* C1 (low SSTw) to an unidentified type of clade D *Symbiodinium* (higher SSTw). *T. reniformis,* as well as *A. millepora,* host D types at locations with high turbidity and in low to medium SST groups, which is not uncommon for D types [67], [68]. Interestingly, *T. reniformis* appears to be relatively hardy or bleaching resistant [40]. It is possible that such host species are more strongly influenced by the lower (winter) limits of locally occurring SST whereas a more bleaching susceptible host species such as *P. damicornis* is more likely to be influenced by the upper (summer) limits of the SST range. While this hypothesis is compelling, direct scientific data in support of it is lacking. Moreover, the *A. millepora* clade D’s in the database were identified as type D1 (pers. comm. T. Pettay) but the type of *Symbiodinium* D hosted by the *T. reniformis* colonies is not known. While *Symbiodinium* types D1 and D1-4 (a.k.a. *Symbiodinium* D1a or *S*. ‘*trenchi’* [9]) are described as “temperature tolerant”, different types of clade D, similar to clade C, are likely to have different ecological, and physiological attributes [19].

Coral host-symbiont combinations often live near their upper temperature tolerance limits implying that the SST at the higher end of the spectrum (i.e. summer SST) would be expected as one of the main driving factors shaping the distribution of species at those locations. Besides the symbiont distribution of *T. reniformis*, SSTs individually influenced that of *S. pistillata* and often contributed to the selection of the best model explaining the *Symbiodinium* distribution in different host species.

Environmental conditions play an important role in structuring the distribution patterns of *Symbiodinium* depending on the spatial scale, i.e. local versus regional comparisons [36], [61]. Combined with ecological speciation and local isolation, all these factors contribute to the biogeographic complexity of host-symbiont associations [9]. While turbidity and SST derived metrics as well as host identity are potential factors driving *Symbiodinium* distributional patterns [9], [34], [35], [36], [61], local adaptation can dampen these effects [69]. As we have shown here, it is unlikely that these different components have a uniform impact across host species. Similarly, environmental effects are unlikely to influence the various host-symbiont combinations in the same way. The absence of other explanatory environmental variables from the model such as depth, irradiance, or nutrient levels could add additional strength when describing the patterns driving symbiont communities. Yet, SST derived variables repeatedly arise as important factors shaping *Symbiodinium* distribution patterns.

### Clade Level Analyses

To reduce dimensionality within the symbiont data set, entries were summed using clade as an indicator. Clade C was very dominant and diverse (56 out of 62 types belonged to clade C) across the GBR, a finding consistent with previous *Symbiodinium* diversity studies demonstrating clade C dominance throughout the Indo-Pacific region [5], [9], [11], [35], [70], [71], [72]. While clades A, B and G were rare, clade D *Symbiodinium* types were found in 6.5% of all host colonies but mostly in conjunction with clade C. Instead of improving the model, clade comparisons significantly decreased the percentage of total variation explained. This is an important result and indicates that differences between *Symbiodinium* clades are not a dominant factor in driving the tolerance and hence distribution of reef symbioses involving *Symbiodinium*. It is also supported by the observation that thermal stress tolerance does not relate to clades [73] and significant differences exist between *Symbiodinium* types of the same clade [26].

It is appealing to interpret the ordination plot as an association with clade D types in high turbidity areas irrespective of SST. While this is partly in agreement with previous findings [61], [74], it is important to bear in mind that the low percentage of total variation indicates the dbRDA axes are of little overall relevance in the multivariate system as a whole. It indicates that other factors, which are not included in the model, likely contribute to overall patterns of variation [60]. In addition, the observed dominance of clade D at high turbidity sites is likely partially driven by host species patterns and local high collection intensity of species such as *A. millepora* and *T. reniformis* that are able to associate with clade D symbionts.

*Symbiodinium* types D1 and D1-4 have previously been related to stressful conditions such as low water quality and high SSTs and may increase the temperature tolerance of the holobiont, but may also function as a putative indicator for weakened coral health [61], [74]. However, there is no evidence that *Symbiodinium* D types other than D1 and D1-4 (e.g. D3, D4-5 and D4-5-9) convey increased temperature tolerance and clade level generalizations should be avoided in this context. In fact D1-4 appears to be an opportunistic species found in the dominant symbiont assemblage of 12 different host species (mostly in hard corals but also in a *Nephthea*) across the GBR and has now been assigned a provisional species name, *Symbiodinium* ‘*trenchi’* [9]. Other clade D types, such as D3 that was found in *Clavularia koellikeri*, present a high degree of host specificity.

### Limitations & General Considerations

There are several limitations that must be taken into account when analyzing and interpreting this dataset or any compiled database aiming to describe what shapes *Symbiodinium* communities across biogeographic scales.

#### Environmental parameters

The environmental parameters used in our analysis reflect long-term time-series. Since some host species may show temporal changes in their host-algae symbiosis [25], [26], [75], the point at which *Symbiodinium* sampling occurred does not necessarily reflect the dominant type through time. However, hosts generally form stable associations with their symbionts and when these associations change, for instance due to environmental disturbance, they often switch back to the previously dominant symbiont type [9], [10], [26], [33], [74]. To avoid picking up temporary dominant symbiont types, sampling efforts that took place directly after bleaching episodes were not included.

The environmental data showed that latitude was a good descriptor of temperature (summer and winter). On the other hand, not all parameters were linked to latitude or longitude and it should be noted that generalized assessments of site characteristics (i.e. in- or offshore reefs) along the GBR are misleading if oceanographic environmental data are not included to form categories. For example, turbidity was not directly linked to the distance from shore. This is likely related to frequent intrusions of clear oceanic waters into the central GBR that tend to flow southwards along the mid-shelf channel, thereby separating the inner and outer reef matrices in the central and southern GBR and promoting variation in turbidity regimes with distance from shore [59]. Additionally, turbidity can be affected by larger tidal ranges in the southern GBR that re-suspend bottom sediments causing increased turbidity [76] or, on more local scales, by various riverine outflows into the GBR (12 major river systems that deposit large sediment plumes). For example, the Burdekin and Fitzroy Rivers are two of the largest GBR riverine systems and are situated in the central and southern sections of the GBR, respectively (Figure 1). As such, inshore reefs in the northern regions could have similar turbidity to more offshore reefs in southern locations due to re-suspended bottom sediments or riverine outputs. Moreover, the complex topography of the broad southern GBR continental shelf influences the current direction resulting in localized oceanographic flow patterns [64].

#### Host sampling

In the establishment of the database it became apparent that: (i) current information on marine invertebrate symbioses involving *Symbiodinium* is concentrated in the central section of the GBR and data are lacking from inshore reefs across all sections, (ii) where information is available the number of sampled host species poorly represents locally present host species assemblages and thus provides an under- as well as skewed estimate of *Symbiodinium* species diversity (Figure 3). Notwithstanding, this meta-analysis includes one of the most extensive genetic datasets on *Symbiodinium* and provides substantial insights into the factors underlying their distribution.

#### *Symbiodinium* identification technique

The database only includes information on the dominant *Symbiodinium* types in the hosts’ symbiont population, omitting data on background *Symbiodinium* obtained by qPCR (picks up approx. <5–10% of the resident *Symbiodinium* population). While in some cases the relative abundance of more tolerant background versus dominant *Symbiodinium* types has been shown to shift upon environmental stress such as increased SST [10], [25] in other cases the symbioses remained stable [77], [78], compromising the ecological relevance of background types to corals in general. In addition non-experimentally treated qPCR data were only available for a few host species on the GBR [32], [36] and background populations of most host colonies included in the database are not known, making it difficult to perform comparative analyses. Excluding background populations from the analysis may, in some cases, underestimate *Symbiodinium* diversity. On the other hand, the low detection levels in qPCR also run the risk of including non-symbiotic types that in fact may obscure environmentally driven patterns. Alternative explanations of the presence of low-density background symbionts may be that they are contamination from the ambient seawater, have unusual ecologies, live on the host mucus or are specialized to live at low densities while others may be transient types that are ingested but fail to form a symbiosis.

### Conclusions

The identity of host species plays a dominant role in determining the distribution of *Symbiodinium.* Although environmental variables explained additional variability, the results were highly host specific. Full comparisons between reefs on longitudinal and latitudinal gradients would be significantly strengthened by inclusion of additional information from targeted locations. Studies aiming to include host-symbiont information to model reef risk and resilience should aim to use greater taxonomic resolution (more detail than clade level) with respect to *Symbiodinium* since it is becoming increasingly evident that intra-cladal differences relate to widely distinct attributes (such as tolerance to temperature or turbidity). In addition, such studies should incorporate the influence of species diversity and host community composition as main driving factors underlying *Symbiodinium* distributional patterns since the two are intrinsically linked. Including the intermixed effects of these processes will improve our understanding of the drivers behind the complexity of reef invertebrate symbioses involving *Symbiodinium* and our ability to generate realistic models estimating the risk of deterioration reefs are exposed to and their resilience in response to a changing climate.

## Supporting Information

Figure S1 **Bubble plots of host abundance data of the eight species (n>100).** Host species include (A) *Acropora millepora*, (B) *A. tenuis*, (C) *A. valida*, (D) *Turbinaria reniformis* (E) *Stylophora pistillata*, (F) *Pocillopora damicornis*, (G) *Seriatopora hystrix*, and (H) *Sinularia flexibilis*. Bubble sizes indicate number of host colonies sampled.
(EPS)

Figure S2 **Principal component analysis (PCO) of the environmental data across sites.** The distribution and designation of (A) SST and (B) turbidity groups and environmental factor projections are shown.
(EPS)

Table S1 **Summary of sampling locations.** Included are: site name, latitude and longitude (in decimal degrees), sea surface temperature (SST) (°C) and turbidity (Z_SD_) groups (SST1∶23.6–24.7; SST2∶25.0–25.7; SST3∶25.8–26.4; SST4∶26.4–27.4; TUR1∶16.3–11.8, TUR2∶11.6–8.8, TUR3∶8.7–5.8, TUR4∶5.0–1.8), # host colonies sampled; # host genera sampled, # host species sampled (hard and octocoral), other host species sampled, # symbiont types, # hard and octocoral species present at each site (transect data extracted from e - atlas), percentage of sampled hard and octocoral species (# host species sampled/# host species from transect data.
(DOCX)

Table S2 **Cross-reference between** ***Symbiodinium*** **types identified with ITS2 and ITS1 rDNA.** Identity for each symbiont ITS type, total # of associated host species and species names are provided.
(DOCX)
