# Correction: Host-Specific Interactions with Environmental Factors Shape the Distribution of *Symbiodinium* across the Great Barrier Reef

**DOI:** 10.1371/annotation/1fb24be5-4809-486e-ae46-0032c2a6c17d

**Published:** 2014-01-16

**Authors:** Linda Tonk, Eugenia M. Sampayo, Scarla Weeks, Marites Magno-Canto, Ove Hoegh-Guldberg

In Table 1 in the online version of the article, the first two rows are missing. Please see the corrected Table 1 here: http://www.plosone.org/corrections/pone.0068533.t001.cn.docx 

