# Supplementary material for: Host-Specific Interactions with Environmental Factors Shape the Distribution of Symbiodinium across the Great Barrier Reef
Source: PLoS One. 2013 Jul 3;8(7):e68533. doi: 10.1371/journal.pone.0068533 (PMC3701053; doi:10.1371/journal.pone.0068533)
Supplement: Table S1 — Summary of sampling locations. Included are: site name, latitude and longitude (in decimal degrees), sea surface temperature (SST) (°C) and turbidity (ZSD) groups (SST1∶23.6–24.7; SST2∶25.0–25.7; SST3∶25.8–26.4; SST4∶26.4–27.4; TUR1∶16.3–11.8, TUR2∶11.6–8.8, TUR3∶8.7–5.8, TUR4∶5.0–1.8), # host colonies sampled; # host genera sampled, # host species sampled (hard and octocoral), other host species sampled, # symbiont types, # hard and octocoral species present at each site (transect data extracted from e - atlas), percentage of sampled hard and octocoral species (# host species sampled/# host species from transect data. (DOCX) [file pone.0068533.s003.docx]

**Table S1** **Summary of sampling locations.**

| **Station** | **Site** | **Lat (°)** | **Long (°)** | **SST group** | **TUR group** | **N (host colonies)** | **Host genera** | **Hard & octocoral spp.** | **Other host spp.** | **Symbiont types** | **Hard & octocoral spp. (e-atlas)** | **% Sampled hard & octocoral spp.** |
| --- | --- | --- | --- | --- | --- | --- | --- | --- | --- | --- | --- | --- |
| 1 | Allonby Island | -20.75 | 149.17 | SST2 | Tur3 | 17 | 1 | 2 | 0 | 2 | 54.05 | 3.70 |
| 2 | Big Broadhurst reef | -18.88 | 147.76 | SST3 | Tur1 | 35 | 2 | 2 | 0 | 2 | 107.15 | 1.87 |
| 3 | Black reef | -19.67 | 149.33 | SST2 | Tur2 | 20 | 1 | 1 | 0 | 1 | 104.95 | 0.95 |
| 4 | Bramble Reef | -18.40 | 146.71 | SST3 | Tur1 | 5 | 4 | 4 | 0 | 3 | 80.55 | 4.97 |
| 5 | Brampton Island | -20.77 | 149.39 | SST2 | Tur3 | 6 | 2 | 2 | 0 | 3 | 56.30 | 3.55 |
| 6 | Bugatti reef | -20.17 | 150.20 | SST2 | Tur1 | 34 | 2 | 2 | 0 | 1 |  |  |
| 7 | Charity Reef | -19.27 | 148.32 | SST3 | Tur1 | 51 | 2 | 3 | 0 | 3 | 104.95 | 2.86 |
| 8 | Chicken Reef | -18.55 | 147.55 | SST3 | Tur1 | 59 | 2 | 3 | 0 | 3 | 91.65 | 3.27 |
| 9 | Credlin Reef | -20.35 | 150.14 | SST2 | Tur3 | 43 | 2 | 3 | 0 | 3 |  |  |
| 10 | Curacao Island | -18.50 | 146.53 | SST3 | Tur1 | 43 | 25 | 39 | 1 | 10 | 58.50 | 66.67 |
| 11 | Darley Reef | -19.17 | 148.15 | SST3 | Tur1 | 60 | 2 | 3 | 0 | 3 | 104.95 | 2.86 |
| 12 | Davies reef | -18.80 | 147.65 | SST4 | Tur1 | 4 | 1 | 1 | 0 | 1 | 107.15 | 0.93 |
| 13 | Daydream Island | -20.27 | 148.83 | SST2 | Tur4 | 18 | 1 | 2 | 0 | 2 | 51.80 | 3.86 |
| 14 | Deloraine Island | -20.16 | 149.04 | SST2 | Tur3 | 19 | 1 | 1 | 0 | 1 | 71.75 | 1.39 |
| 15 | Dingo Reef | -19.17 | 148.38 | SST3 | Tur1 | 44 | 2 | 3 | 0 | 3 | 104.95 | 2.86 |
| 16 | Double Cone Island | -20.10 | 148.72 | SST2 | Tur3 | 1 | 1 | 0 | 1 | 1 | 67.25 | 0.00 |
| 18 | Elford reef | -16.92 | 146.29 | SST4 | Tur1 | 36 | 2 | 2 | 0 | 2 | 65.10 | 3.07 |
| 19 | Fantome Island | -18.68 | 146.51 | SST2 | Tur3 | 31 | 5 | 6 | 0 | 2 | 71.75 | 8.36 |
| 20 | Faraday Reef | -18.42 | 147.35 | SST4 | Tur1 | 17 | 7 | 7 | 0 | 4 | 76.15 | 9.19 |
| 21 | Feather reef north | -17.52 | 146.39 | SST4 | Tur2 | 124 | 49 | 79 | 5 | 21 | 67.30 | 117.38 |
| 22 | Feather reef south | -17.53 | 146.39 | SST4 | Tur2 | 21 | 17 | 14 | 3 | 9 | 67.30 | 20.80 |
| 23 | Flora reef | -17.18 | 146.29 | SST4 | Tur1 | 35 | 2 | 2 | 0 | 2 | 67.30 | 2.97 |
| 24 | Girder Reef | -19.72 | 149.37 | SST2 | Tur2 | 20 | 2 | 2 | 0 | 2 | 104.95 | 1.91 |
| 25 | Grassy Island | -20.13 | 148.61 | SST2 | Tur4 | 20 | 1 | 1 | 0 | 1 | 67.25 | 1.49 |
| 26 | Great Palms Cannon Bay | -18.77 | 146.56 | SST4 | Tur3 | 8 | 3 | 3 | 0 | 2 | 73.95 | 4.06 |
| 27 | Great Palms Channel | -18.67 | 146.56 | SST3 | Tur2 | 34 | 4 | 4 | 0 | 3 | 73.95 | 5.41 |
| 29 | Hazelwood | -20.27 | 149.11 | SST2 | Tur3 | 1 | 1 | 0 | 1 | 1 | 71.75 | 0.00 |
| 30 | Herald Prong reef | -21.55 | 151.39 | SST2 | Tur2 | 35 | 2 | 2 | 0 | 2 |  |  |
| 31 | Heron Isl., points | -23.44 | 151.92 | SST1 | Tur2 | 231 | 36 | 82 | 3 | 29 |  |  |
| 32 | Heron Island, channel | -23.44 | 151.90 | SST1 | Tur4 | 505 | 10 | 11 | 0 | 17 |  |  |
| 36 | Heron Island, north | -23.44 | 151.94 | SST1 | Tur2 | 224 | 5 | 5 | 0 | 12 |  |  |
| 37 | Hook Is. North | -20.09 | 148.90 | SST3 | Tur3 | 3 | 2 | 2 | 0 | 2 | 71.75 | 2.79 |
| 38 | John Brewer Reef | -18.63 | 147.05 | SST3 | Tur2 | 18 | 10 | 10 | 0 | 5 | 93.85 | 10.66 |
| 39 | Keswick Island | -20.90 | 149.43 | SST2 | Tur3 | 10 | 1 | 1 | 0 | 2 | 38.60 | 2.59 |
| 41 | Lodestone Reef | -18.69 | 147.11 | SST3 | Tur3 | 13 | 5 | 6 | 0 | 4 | 93.85 | 6.39 |
| 42 | Long Island | -20.35 | 148.85 | SST2 | Tur4 | 3 | 2 | 2 | 0 | 2 | 51.80 | 3.86 |
| 44 | Keppel Isl., Miall | -23.15 | 150.90 | SST1 | Tur4 | 477 | 1 | 2 | 0 | 4 | 34.19 | 5.85 |
| 45 | Mumford Island | -21.98 | 149.85 | SST1 | Tur4 | 9 | 1 | 1 | 0 | 1 | 34.19 | 2.93 |
| 46 | Magnetic Isl., Nelly Bay | -19.15 | 146.85 | SST3 | Tur4 | 97 | 4 | 12 | 0 | 4 | 65.09 | 18.44 |
| 47 | Noreaster reef | -17.77 | 146.64 | SST4 | Tur1 | 39 | 2 | 2 | 0 | 2 | 62.90 | 3.18 |
| 48 | Keppel Island, north | -23.08 | 150.88 | SST1 | Tur4 | 49 | 3 | 3 | 0 | 3 | 34.19 | 8.78 |
| 49 | One Tree Island | -23.50 | 152.08 | SST1 | Tur2 | 112 | 9 | 11 | 0 | 8 |  |  |
| 50 | Orpheus Island | -18.58 | 146.49 | SST3 | Tur3 | 68 | 1 | 1 | 4 | 4 | 56.30 | 1.78 |
| 51 | Paul reef | -21.35 | 150.83 | SST2 | Tur2 | 2 | 1 | 1 | 0 | 1 |  |  |
| 52 | Pelorus island | -18.57 | 146.50 | SST3 | Tur2 | 1 | 1 | 1 | 0 | 1 | 56.30 | 1.78 |
| 53 | Percy Island | -21.67 | 150.41 | SST2 | Tur2 | 38 | 2 | 2 | 0 | 2 | 51.80 | 3.86 |
| 54 | Phillips Reef | -21.47 | 149.50 | SST1 | Tur4 | 6 | 2 | 2 | 0 | 2 | 36.35 | 5.50 |
| 55 | Pickersgill reef | -15.87 | 145.58 | SST4 | Tur2 | 33 | 2 | 2 | 0 | 2 | 62.90 | 3.18 |
| 57 | Rib reef north | -18.47 | 146.87 | SST3 | Tur1 | 79 | 42 | 59 | 5 | 22 | 96.05 | 61.43 |
| 58 | Rib reef south | -18.50 | 146.86 | SST3 | Tur2 | 47 | 25 | 34 | 6 | 14 | 96.05 | 35.40 |
| 59 | Ribbon reef | -15.33 | 145.79 | SST4 | Tur1 | 24 | 2 | 2 | 0 | 2 | 60.70 | 3.29 |
| 60 | Stone Island | -20.04 | 148.28 | SST2 | Tur4 | 20 | 1 | 2 | 0 | 2 | 65.09 | 3.07 |
| 61 | Trunk Reef | -18.40 | 146.82 | SST3 | Tur2 | 5 | 1 | 3 | 0 | 2 | 80.55 | 3.72 |
| 62 | UN 19-165 | -19.78 | 149.47 | SST2 | Tur2 | 2 | 1 | 1 | 0 | 1 | 104.95 | 0.95 |
| 63 | UN 21-056 | -19.80 | 149.47 | SST2 | Tur4 | 17 | 2 | 3 | 0 | 3 | 104.95 | 2.86 |
| 64 | Magnetic Isl., West Point | -19.13 | 146.75 | SST3 | Tur4 | 14 | 1 | 1 | 0 | 2 | 65.09 | 1.54 |
| 65 | Wistari reef | -23.50 | 151.88 | SST1 | Tur4 | 18 | 1 | 1 | 0 | 1 |  |  |
| 66 | Deloraine Is east | -20.15 | 149.07 | SST2 | Tur3 | 30 | 1 | 1 | 0 | 1 | 71.75 | 1.39 |
| 68 | Hook Is west | -20.12 | 148.88 | SST2 | Tur3 | 30 | 1 | 1 | 0 | 1 | 71.75 | 1.39 |
| 69 | Orpheus Is (N channel) | -18.57 | 146.48 | SST3 | Tur2 | 30 | 1 | 1 | 0 | 1 | 56.30 | 1.78 |
| 70 | Rib Reef | -18.48 | 146.87 | SST3 | Tur4 | 30 | 1 | 1 | 0 | 1 | 96.05 | 1.04 |
| 71 | Trunk Reef, channel | -18.35 | 146.77 | SST3 | Tur3 | 30 | 1 | 1 | 0 | 1 | 80.55 | 1.24 |
| 72 | Agincourt Reef | -15.98 | 145.82 | SST4 | Tur1 | 30 | 1 | 1 | 0 | 1 | 47.45 | 2.11 |
| 73 | Day reef | -14.47 | 145.56 | SST4 | Tur1 | 144 | 1 | 1 | 0 | 5 |  |  |
| 74 | Escape Reef | -15.82 | 145.80 | SST4 | Tur1 | 30 | 1 | 1 | 0 | 1 | 45.25 | 2.21 |
| 75 | Lizard Island | -14.68 | 145.48 | SST3 | Tur1 | 90 | 3 | 3 | 0 | 4 | 78.35 | 3.83 |
| 77 | Undine Reef | -16.10 | 145.63 | SST4 | Tur2 | 30 | 1 | 1 | 0 | 1 | 62.90 | 1.59 |
| 78 | Yonge reef | -14.60 | 145.66 | SST4 | Tur3 | 204 | 1 | 1 | 0 | 3 |  |  |

Included are: site name, latitude and longitude (in decimal degrees), sea surface temperature (SST) (°C) and turbidity (Z_SD_) groups (SST1: 23.6 – 24.7; SST2: 25.0 – 25.7; SST3: 25.8 – 26.4; SST4: 26.4 – 27.4; TUR1: 16.3 – 11.8, TUR2: 11.6 – 8.8, TUR3: 8.7 – 5.8, TUR4: 5.0 – 1.8), # host colonies sampled; # host genera sampled, # host species sampled (hard and octocoral), other host species sampled, # symbiont types, # hard and octocoral species present at each site (transect data extracted from e - atlas), percentage of sampled hard and octocoral species (# host species sampled/# host species from transect data. (DOCX)
