# Supplementary material for: Host-Specific Interactions with Environmental Factors Shape the Distribution of Symbiodinium across the Great Barrier Reef
Source: PLoS One. 2013 Jul 3;8(7):e68533. doi: 10.1371/journal.pone.0068533 (PMC3701053; doi:10.1371/journal.pone.0068533)
Supplement: Table S2 — Cross-reference between Symbiodinium types identified with ITS2 and ITS1 rDNA. Identity for each symbiont ITS type, total # of associated host species and species names are provided. (DOCX) [file pone.0068533.s004.docx]

**Table S2** **Cross-reference between *Symbiodinium* types identified with ITS2 and ITS1 rDNA.**

| ***ITS1 Type*** | ***ITS2 Type*** | ***# Host***  ***species*** | ***Host species*** |
| --- | --- | --- | --- |
|  | A | 3 | *Acropora longicyathus, Acropora millepora, Acropora valida* |
|  | A/C3 | 1 | *Acropora valida* |
|  | A/C3/C1 | 1 | *Acropora valida* |
|  | A7 | 1 | *Millepora platyphylla* |
|  | B1 | 1 | *Nephthea sp.* |
|  | B36 | 1 | *Nephthea sp.* |
|  | C | 4 | *Alertigorgia orientalis, Briareum violacea, Acropora longicyathus, Seriatopora hystrix* |
|  | C/D | 2 | *Acropora tenuis, Acropora valida* |
| C1, C1:1a; C1:2; C1:3a | C1 | 69 | *Lobophytum compactum, Lobophytum sp., Rhytisma sp., Sarcophyton sp., Sinularia flexibilis, Sinularia polydactyla, Sinularia sp., Icilogorgia, Briareum sp., Pinnigorgia flava, Rumphella sp., Plumigorgia sp., Lemnalia sp., Paralemnalia digitiformis, Paralemnalia thyrsoidea, Efflatounaria sp., Paralemnalia digitiformis, Corallimorpharia sp., Discosoma sp., Millepora exaesa, Heliopora coerulea, Acropora aculeus, Acropora cerealis, Acropora millepora, Acropora secale, Acropora tenuis, Astreopora myriophthalmata, Leptoseris yabei, Pavona duerdeni, Euphyllia divisa, Plerogyra sinuosa, Turbinaria reniformis, Turbinaria stellulata, Cyphastrea decadia, Cyphastrea microphthalma, Cyphastrea serailia, Favia pallida, Favia speciosa, Goniastrea australensis, Goniastrea pectinata, Fungia fungites, Fungia granulosa, Fungia sp., Herpolitha weberi, Polyphyllia talpina, Merulina ampliata, Merulina scabricula, Scolymia australis, Galaxea astreata, Galaxea fascicularis, Echinophyllia echinoporoides, Mycedium elephantotus, Pocillopora damicornis, Stylophora pistillata, Alveopora fenestrata, Goniopora djiboutiensis, Goniopora minor, Goniopora tenuidens, Coscinaraea columna, Leptastrea pruinosa, Leptastrea purpurea, Psammacora cartigua, Psammocora contigua, Psammocora digitata, Psammocora profundacella, Tridacna derasa, Tridacna gigas, Tridacna maxima, Palythoa sp.* |
|  | C1/A | 1 | *Pocillopora damicornis* |
|  | C1/B36 | 1 | *Nephthea sp.* |
|  | C1/C3 | 5 | *Acropora florida, Acropora humilis, Acropora longicyathus, Acropora millepora, Acropora tenuis* |
|  | C1/C3/D | 1 | *Acropora millepora* |
|  | C1/D | 2 | *Turbinaria reniformis, Pocillopora damicornis* |
|  | C1/D1 | 1 | *Acropora nobilis* |
|  | C120 | 1 | *Seriatopora hystrix* |
|  | C120a | 1 | *Seriatopora hystrix* |
| Cdot | C15 | 15 | *Stereonephthya sp., Asterospicularia laurae, Cespitularia sp., Heteroxenia sp., Xenia sp., Aglaeophenia sp., Montipora digitata, Porites annae, Porites cylindrica,*  *Porites lichen, Porites mayeri, Porites nigrescens, Porites rus, Porites sp., Porites vaughani* |
|  | C15/C73 | 1 | *Montipora digitata* |
|  | C15e | 1 | *Millepora tenella* |
| Cdot | C17 | 2 | *Montipora aequituberculata, Montipora monasteriata* |
|  | C17/C15 | 7 | *Montipora aequituberculata, Montipora crassituberculata , Montipora digitata, Montipora hispida, Montipora mollis , Montipora peltiformis, Montipora spongodes* |
|  | C1b | 3 | *Tubipora musica, Pavona varians, Leptastrea purpurea* |
|  | C1c | 6 | *Sinularia sp., Efflatounaria sp., Pocillopora damicornis, Pocillopora eydouxi, Pocillopora meandrina, Pocillopora verrucosa* |
|  | C1c* | 1 | *Efflatounaria sp.* |
|  | C1d | 1 | *Cyphastrea serailia* |
|  | C1j | 1 | *Pocillopora damicornis* |
|  | C1k | 1 | *Xenia sp.* |
|  | C1m-aa | 1 | *Seriatopora hystrix* |
|  | C1q | 1 | *Cespitularia sp.* |
|  | C21 | 16 | *Acropora latistella, Montipora foveolata, Leptoseris explanata, Pachyseris speciosa, Pavona explanulata, Echinopora lamellosa, Favia favus, Favites abdita, Favites halicora,*  *Goniastrea favulus, Fungia fungites, Fungia sp., Merulina ampliata, Lobophyllia corymbosa, Galaxea achelia, Echinophyllia aspera* |
|  | C22a | 2 | *Turbinaria heronensis, Turbinaria peltata* |
|  | C22a/C3 | 1 | *Lobophyllia corymbosa* |
|  | C23 | 2 | *Briareum sp., Isis sp.* |
|  | C24 | 1 | *Palythoa sp.* |
|  | C25 | 1 | *Heteractis magnifica* |
|  | C26 | 1 | *Montipora stellata* |
|  | C26a | 3 | *Montipora hispida, Montipora monasteriata, Montipora turtlensis* |
|  | C27 | 1 | *Pavona varians* |
|  | C28 | 1 | *Porites annae* |
| C2 | C3 | 65 | *Isis sp., Acropora austera, Acropora cerealis, Acropora clathrata, Acropora cytherea, Acropora danei, Acropora digitifera, Acropora formosa, Acropora gemmifera, Acropora grandis, Acropora humilis, Acropora hyacinthus, Acropora latistella, Acropora longicyathus, Acropora loripes, Acropora microclados, Acropora millepora, Acropora monticulosa, Acropora nasuta, Acropora nobilis, Acropora palifera, Acropora polystoma, Acropora sarmentosa, Acropora secale, Acropora subulata, Acropora tenuis, Acropora torresiana, Acropora valida, Acropora yongei, Montipora aequituberculata, Caulastrea furcata, Cyphastrea serailia, Diploastrea heliopora, Echinopora gemmacea, Echinopora lamellosa, Favia rotundata, Favia stelligera, Favites abdita, Favites complanata, Goniastrea aspera, Goniastrea australensis, Goniastrea edwardsi, Goniastrea favulus, Goniastrea pectinata, Goniastrea retiformis, Leptoria phrygia, Montastrea annuligera, Montastrea curta, Montastrea valenciennesi, Platygyra daedalea, Platygyra lamellina, Platygyra pini, Hydnophora exesa, Merulina ampliata, Acanthastrea echinata, Lobophyllia corymbosa, Lobophyllia hemprichii, Lobophyllia sp., Symphyllia agaricia, Symphyllia radians, Echinophyllia mammiformis, Mycedium elephantotus, Seriatopora hystrix, Palythoa sp., T. reniformis* |
|  | C3-ff | 1 | *Seriatopora hystrix* |
|  | C3/A | 1 | *Acropora valida* |
|  | C3/C1 | 1 | *Acropora valida* |
|  | C3/C1/A | 1 | *Acropora valida* |
|  | C3/D | 1 | *Acropora millepora* |
|  | C3/D1 | 1 | *Acropora palifera* |
|  | C3/D1a | 3 | *Leptoria phrygia, Montastraea curta, Hydnophora microconos* |
|  | C31 | 1 | *Montipora turtlensis* |
|  | C31b | 1 | *Alveopora fenestrata* |
|  | C33 | 1 | *Pocillopora damicornis* |
|  | C33a | 1 | *Pocillopora damicornis* |
|  | C35a | 1 | *Stylophora pistillata* |
|  | C35a/C78a | 1 | *Stylophora pistillata* |
|  | C3h | 39 | *Acropora aculeus, Leptoseris yabei, Pachyseris rugosa, Pachyseris speciosa, Pavona explanulata, Pavona maldivensis, Turbinaria frondens, Caulastrea furcata, Echinopora gemmacea, Echinopora hirsutissima, Favites abdita, Goniastrea pectinata, Oulophyllia crispa, Platygyra daedalea, Platygyra pini, Platygyra ryukyuensis, Fungia echinata, Fungia fungites, Fungia granulosa, Fungia horrida, Fungia paumotensis, Fungia sp., Heliofungia actiniformis, Lithophyllon undulatum, Podabacia crustacea, Sandalolitha robusta, Hydnophora exesa, Lobophyllia sp., Scolymia australis, Echinophyllia aspera, Echinophyllia echinata, Echinophyllia orpheensis, Mycedium elephantotus, Oxypora glabra, Oxypora lacera, Pectinia lactuca, Pectinia paeonia, Pectinia sp., Leptastrea pruinosa* |
|  | C3h/C1 | 1 | *Polyphyllia talpina* |
|  | C3ha | 4 | *Gardineroseris planulata, Lobophyllia hemprichii, Echinophyllia aspera, Oxypora glabra* |
|  | C3i | 7 | *Acropora cerealis, Acropora digitifera, Acropora florida, Acropora gemmifera, Acropora humilis, Acropora nasuta, Acropora nobilis* |
|  | C3j | 4 | *Lobophytum sp., Sarcophyton sp., Sinularia sp., Acropora tenuis* |
|  | C3k | 13 | *Acropora cerealis, Acropora cytherea, Acropora digitifera, Acropora divaricata, Acropora florida, Acropora grandis, Acropora humilis, Acropora hyacinthus,*  *Acropora millepora, Acropora monticulosa, Acropora nasuta, Acropora nobilis, Acropora palifera* |
|  | C3n-t | 1 | *Seriatopora hystrix* |
|  | C40 | 7 | *Turbinaria frondens, Turbinaria reniformis, Echinopora hirsutissima, Echinopora lamellosa, Symphyllia radians, Symphyllia recta, Mycedium elephantotus* |
|  | C40a | 1 | *Echinopora horrida* |
|  | C42 | 1 | *Pocillopora damicornis* |
|  | C42a | 1 | *Pocillopora damicornis* |
|  | C42ab | 1 | *Pocillopora damicornis* |
|  | C42b | 1 | *Pocillopora damicornis* |
|  | C60 | 3 | *Porites annae, Porites cylindrica, Porites vaughani* |
|  | C61 | 1 | *Montipora grisea* |
|  | C62 | 1 | *Zoanthus sp.* |
|  | C64 | 6 | *Klyxum sp., Lemnalia sp., Paralemnalia digitiformis, Anthelia sp., Heteroxenia sp., Xenia sp.* |
|  | C65 | 4 | *Lobophytum sp., Sarcophyton sp., Sinularia sp., Hicksonella sp.* |
|  | C67 | 1 | *Heteractis magnifica* |
|  | C68 | 1 | *Heteractis magnifica* |
|  | C69a | 1 | *Heteractis crispa* |
|  | C78a | 1 | *Stylophora pistillata* |
|  | C79 | 1 | *Stylophora pistillata* |
|  | C8 | 1 | *Stylophora pistillata* |
|  | C84a | 1 | *Anthelia sp.* |
| C1n | C8a | 1 | *Stylophora pistillata* |
|  | C8a/G | 1 | *Stylophora pistillata* |
|  | D | 4 | *Alertigorgia orientalis, Clavularia sp., Tubipora musica, Acropora millepora* |
|  | D1 | 3 | *Acropora aculeus, Acropora palifera, Acropora paniculata* |
|  | D1-4 | 8 | *Nephthea sp., Echinopora hirsutissima, Favia sp., Goniastrea favulus, Acanthastrea echinata, Galaxea astreata, Galaxea fascicularis, Galaxea horrescens* |
|  | D3 | 2 | *Clavularia koellikeri, Clavularia sp.* |
|  | G | 5 | *Cliona caesia, Cliona orientalis, S. pistillata, E. nuttingi, J. fragilis* |

Identity for each symbiont ITS type, total # of associated host species and species names are provided. (DOCX)
